# Comparison of Ultrasonic Welding and Thermal Bonding for the Integration of Thin Film Metal Electrodes in Injection Molded Polymeric Lab-on-Chip Systems for Electrochemistry

**DOI:** 10.3390/s16111795

**Published:** 2016-10-27

**Authors:** Marco Matteucci, Arto Heiskanen, Kinga Zór, Jenny Emnéus, Rafael Taboryski

**Affiliations:** Department of Micro- and Nanotechnology, Technical University of Denmark, Kongens Lyngby 2800, Denmark; arto.heiskanen@nanotech.dtu.dk (A.H.); kinzo@nanotech.dtu.dk (K.Z.); jenny.emneus@nanotech.dtu.dk (J.E.); rata@nanotech.dtu.dk (R.T.)

**Keywords:** ultrasonic welding, metal electrodes, injection molding, electrochemistry, microfluidics

## Abstract

We compare ultrasonic welding (UW) and thermal bonding (TB) for the integration of embedded thin-film gold electrodes for electrochemical applications in injection molded (IM) microfluidic chips. The UW bonded chips showed a significantly superior electrochemical performance compared to the ones obtained using TB. Parameters such as metal thickness of electrodes, depth of electrode embedding, delivered power, and height of energy directors (for UW), as well as pressure and temperature (for TB), were systematically studied to evaluate the two bonding methods and requirements for optimal electrochemical performance. The presented technology is intended for easy and effective integration of polymeric Lab-on-Chip systems to encourage their use in research, commercialization and education.

## 1. Introduction

One of the major issues in the assembly of polymeric micro- and nanofluidic systems is proper sealing of the devices [1]. This is particularly true when the bonding of thermoplastic devices is required on a large scale. Several bonding techniques have been proposed for thermoplastics, the most common being solvent bonding [2], laser welding [3], adhesive bonding [4], and thermal bonding (TB) [5,6]. The bonding step is particularly critical in Lab-on-Chip (LoC) systems with integrated electrodes, the performance of which needs to be maintained while achieving a completely leakage-free system. At present, many electrode materials, such as polymers [7], metals [8], carbon nanotubes [9], and graphene [10] have been successfully implemented and used in academic applications. Although the integration of polymers with large-scale fabrication techniques have given remarkable results [11,12], a method of bonding microfluidic chips with integrated electrodes effectively and compatibly with production standards is yet to be found. 

Ultrasonic welding (UW) [1,13,14] is an extremely appealing technique for the bonding of polymers, since it is fast (a few seconds per sample), and since localized UW eliminates the need for potentially hazardous solvents and high temperatures. For these reasons, UW is compatible with the pre-loading of temperature-sensitive materials or even of biological material, such as DNA [15]. Recently, UW has been used (amongst other techniques) for the assembly of piezo micropumps [16], to perform polymerase chain reaction (PCR) [17], and in combination with TB [18,19,20]. 

**Figure 1:**
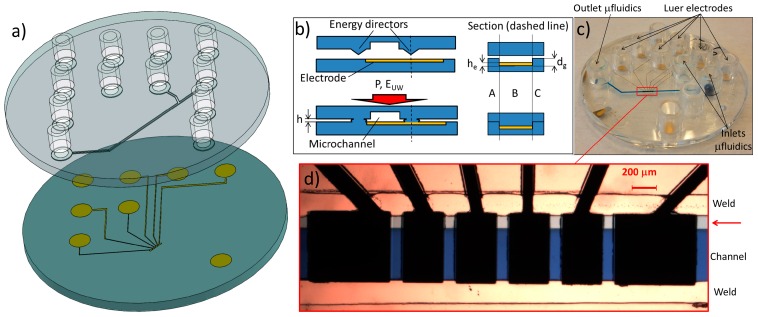
(**a**) 3D scheme of the chip prior to thermal bonding (TB): on the top, the fluidics includes 12 standard Luer fittings for easy connection to external pumping. The only difference between the design for TB and ultrasonic welding (UW) is the presence of energy directors surrounding the fluidic channel. The bottom substrate is used to embed six electrodes; (**b**) Schematic presentation of UW bonding of chips with embedded electrodes. The pressure P and energy E_UW_ are optimized to seal the channel, preserve the electrodes, and minimize the added height h. In cross-section, the behaviour of the energy directors is shown: while the melting in sections A and C allows the welding, the lower amount of energy dispersed in B (where energy directors are not in contact with the substrate) allows the filling of the void with depth d_g_-h_e_ without damaging the electrodes; (**c**) Cyclic olefin copolymer (COC) chip with 12 Luer fittings bonded with UW (blue dye demonstrates successful sealing of the microfluidic channel); (**d**) Details of the six welded electrodes. The outermost ones (650 μm × 550 μm) are used as reference (RE) and counter (CE) electrodes, while the four innermost ones (325 μm × 550 μm) are working electrodes (WE). Note how the added height h causes a small dead volume (red arrow). The latter is identified by a shadowing caused by the expansion of a small quantity of blue dye.

During the UW process, welding occurs when ultrasound energy—applied for a few hundred milliseconds—is concentrated on specifically-designed structures (called energy directors) on a sample that is pressed against a substrate. The ultrasound energy makes the energy directors melt, allowing bonding of the two parts (Figure 1a,b). It was demonstrated that UW is a very useful tool for both self-aligned gapless welding of polymer chips and for sealing of low-aspect ratio, large-area microchannels [1,13,14,16,21,22]. Here, we focus on the use of UW for the integration of embedded metal electrodes in injection molded (IM) microfluidic chips (Figure 1b,c) made of cyclic olefin copolymer (COC). The use of UW to weld COC-based polymer systems with electrodes was previously suggested by Naseri et al. [23]. However, they neither gave any details on the welding quality, nor provided any information regarding the geometry of the energy directors. Moreover, Naseri et al. presented IM electrodes (2 or 5 mm wide and several mm long) made of carbon-loaded COC, coated one-by-one with different thin metal films used as electrodes and embedded in the fluidics by means of an over-molding process. The major limitations of such a process are that it is not possible to make micron-sized electrodes, and that the manual insertion of the electrodes heavily limits the scalability of production throughput. 

Here we describe a highly-scalable fabrication method to make embedded microelectrodes that are then coupled to fluidics either by TB or UW. The key to our fabrication method is a surface roughness of the IM samples that can be as low as 2 nm inside the carved microchannels [24,25]. A roughness that is three orders of magnitude smaller than the energy directors allows the concentration of the ultrasonic energy, and hence, bonding using a lower amount of energy compared to rougher samples that can cause energy dispersion on the substrate surface. For this reason, the energy directors can be lower than in the case of substrates with higher surface roughness, such as the ones obtained by replicating stamps made by direct milling techniques [21]. Moreover, the use of UW for the bonding of chips made by a high-throughput technique such as IM opens new possibilities for mass fabrication of electrochemical systems. 

## 2. Materials and Methods

**Figure 2:**
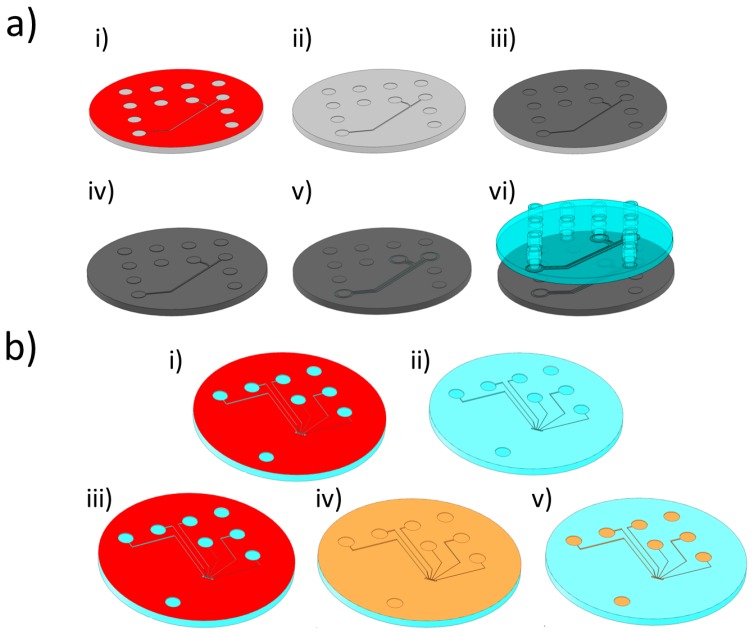
(**a**) Stamp (here referred to as shim) fabrication and polymer injection molding of microfluidic chips: (i) UV lithography on Si wafer; (ii) Deep Reactive Ion Etching of Si and resist stripping; (iii) Sputtering of metal seed layer; iv) Ni electroplating and Si removal in potassium hydroxide; (v) Laser ablation of shim for engraving of energy director template (only for ultrasonic welded samples); (vi) injection molding of TOPAS chips; (**b**) Electrode fabrication on injection molded TOPAS flats: (i) UV lithography on TOPAS; (ii) Reactive Ion Etching of grooves; (iii) Aligned lithography with resist image reversal. The lithography was performed with features slightly smaller than the ones used for making grooves, so as to entirely embed the electrodes inside the substrate; (iv) E-beam evaporation of metal layers; (v) Resist lift-off in acetone.

The electrodes and microfluidic design used for both bonding techniques were the same. The microfluidic design consists of 80 μm deep, 400 μm wide channels. The only difference between the microchannel designs for TB and UW was that substrates for the latter included energy directors. IM of the substrates was done using a Ni stamp (shim) fabricated by standard cleanroom processing techniques, as illustrated in Figure 2a and described in greater detail elsewhere [26]. To generate energy directors on the IM substrates, triangular grooves were patterned on the Ni shim around the microfluidic channel templates (Figure 2a(v)) by laser ablation (microSTRUCT vario, 3D-Micromac AG, Chemnitz, Germany). Five parallel lines were patterned with a distance of 20 μm between them to obtain the triangular geometry [27]. By changing the number of iterations of the laser ablation, energy director areas with different sections (base: 100 μm; height: 8, 32, or 65 μm) were fabricated. In order to minimize the energy delivered during UW (and thus the chance of damaging the electrodes), the 8 μm-high energy directors were chosen. Both substrates—one with embedded electrodes and another containing the fluidics—were 2 mm-thick discs (∅ = 50 mm) made in COC (TOPAS 5013L-10, TOPAS Advanced Polymers GmbH, Frankfurt am Main, Germany) by IM. The bottom substrates comprised six electrodes (Figure 1c,d): four working (WE), one reference (RE) and one counter (CE) electrode. The electrodes were embedded inside square grooves to protect them from the damage caused by the mechanical or thermal stress induced during UW and TB [28].

Both bonding techniques can potentially damage the electrodes: while TB can cause stress to the electrodes due to the difference in thermal expansion coefficients of TOPAS and metal, UW can seriously damage the electrical connection because of the high delivered energy. The square grooves were made with varying depths (*d*_g_), ranging from 900 nm to 5 μm. Electrodes with no grooves were also fabricated for comparison. The electrode fabrication procedure is described in Figure 2b. The grooves were made on the substrates by creating a masking layer of AZ resist, patterning it with UV-lithography, and then etching the TOPAS by reactive ion etching (RIE) [28]. Anisotropy of the etching was assured by keeping the power of the radiofrequency at 150 W and thus obtaining high-energy ions inside the oxygen plasma. The thickness of the masking layer was kept high (4.2–10 μm) due to the poor selectivity of the AZ resist with respect to TOPAS. When optimizing the etching recipe for the deepest grooves (groove depth *d*_g_ = 5 μm), we measured a selectivity <0.5:1. After removing the resist in acetone, a second aligned lithography step was required to define the embedded electrode structures, leads, and contact pads inside the grooves. A 1.5 μm-thick film of AZ5412E was deposited and exposed before a reversal bake (25 min in oven at 120 °C), flood exposure (30 s @ 7 mW/cm^2^), and development (60 s) were performed [29]. Evaporation of a Ti/Au bi-layer (10 nm Ti and 200 nm Au) was followed by a resist lift-off in acetone to finalize electrode fabrication. The yield of the lift-off process was very near to 100%, due to the extreme flatness of the substrates. The latter were made by IM of stamps fabricated by Ni electroplating of a bare Si wafer.

In order to simplify the fabrication, a procedure to make a template for the electrode grooves directly on the stamp was implemented. Substrates with pre-molded grooves were fabricated. Unfortunately, the alignment of the molded grooves with the original electrode pattern was not possible because of the substrate contraction after IM. Such a contraction is due to the polymer shrinkage in the molding process. Considering a substrate diameter of 50 mm, a 100 K temperature change between the mold and room temperature, together with a coefficient of linear thermal expansion of 6 × 10^−5^ K^−1^ [30], the calculated contraction due to cooling is around 300 μm. This value is compatible with the ones resulting from the misalignment we measured between the molded alignment marks and the ones present on the mask for the second lithography. A further issue is the difference in contraction geometry of fluidic and electrode substrate due to the different patterns. In order to design grooves for optimal alignment, an in-depth study of the contraction is needed. For this reason, the IM of the electrode grooves was abandoned. The final microfluidic chips were obtained by aligning the substrates with fluidic channels to the ones with embedded electrodes and performing the bonding. For external connection to fluidics, the top part was also equipped with 12 Luer connectors [5] (Figure 1a,c). 

UW was performed with a 20 kHz Telsonic USP4700 welder (Telsonic GmbH, Erlangen, Germany) that includes an in-house built holder with housings to protect the Luer fittings during bonding. To obtain optimal channel sealing and low stress to the electrodes, the delivered energy (*E*_UW_) was varied in the UW tests. Other UW parameters (such as trigger force, F_u_, and transducer amplitude, A) are listed in Table 1. To enhance TB, both electrode and fluidic substrates were exposed to UV (DYMAX mercury UV-bulb F/5000, Dymax Corporation, Torrington, CT, USA) for 30 s. TB was performed with a manual press (P/O/Weber, Paul-Otto Weber Maschinen und Apparatebau GmbH, Remshalden, Germany) with decoupled plate temperature control and force controller (P/O/Weber Presstronic, Paul-Otto Weber Maschinen und Apparatebau GmbH, Remshalden, Germany). To eliminate non-parallelisms of the press plates, a 2 mm-thick polydimethylsiloxane (PDMS) layer was put on top of the samples prior to bonding. The bonding temperature (*T*), force (*F*_t_), and bonding time (*t*) were varied during TB optimization (Table 1). The bonding energy (*G*_f_) of the TB chips was calculated by measuring the crack length (*L*) and the razor blade method [26,31]:

$$G_{f}=\frac{3Ed^{3}\delta^{2}}{2L^{4}}$$

where *E* and *d* are, respectively, the plate modulus and the thickness of the polymer substrate, while *δ* is the blade thickness. The characterization of the UW chips was performed by leak test. Characterization of delaminated samples was performed both by scanning electron microscopy (Supra 40VP SEM, Carl Zeiss Jena GmbH, Jena, Germany) and mechanical profilometry (Dektak XTA stylus profiler, Bruker Daltonics Inc., Billerica, MA, USA).

Electrochemical characterization was performed using phosphate buffered saline (PBS, pH = 7.4) containing 10 mM potassium hexacyanoferrate(III/II). All of the chemicals were purchased from Sigma-Aldrich Corporation (St. Louis, MO, USA), and the solutions were prepared using ultrapure water (resistivity 18.2 MΩ·cm) from a Milli-Q^®^ water purification system (Millipore Corporation, Billerica, MA, USA).

For electrochemical measurements, the microfluidic channel of each chip was manually filled with electrolyte solution using a syringe placed in one of the Luer connectors leading to the channel. For characterization purposes, electrical resistance measurements of pairs of neighboring electrodes were performed. The minimum expected resistance value is the solution resistance which was initially measured with an open-well system to be around 100 Ω. When the four on-chip working electrodes (WE) were characterized, the larger on-chip electrodes were used as a counter (CE) and reference (RE) electrodes. All the electrochemical tests were performed on electrodes as-fabricated without need for prior cleaning. Cyclic voltammetric (CV) characterization was performed using a 1010 A potentiostat from CH Instruments, Inc. (Austin, TX, USA) in a potential window between −300 and 300 mV at the scan rate of 100 mV/s. Electrochemical impedance spectroscopic (EIS) characterization was performed using a Reference 600 potentiostat from Gamry Instruments (Warminster, PA, USA) operated by EIS300 software (v. 6.10). The sinusoidal perturbation potential (10 mV rms) was applied with respect to the open circuit potential in the frequency range between 1 Hz and 100 kHz. Data analysis was done using EchemAnalyst software (v. 6.10) from Gamry Instruments by fitting the data to Randles equivalent circuit model for electrical impedance Z using nonlinear least-squares (NLLS) regression [32,33].

## 3. Results

### 3.1. Bonding Characterization

**Figure 3:**
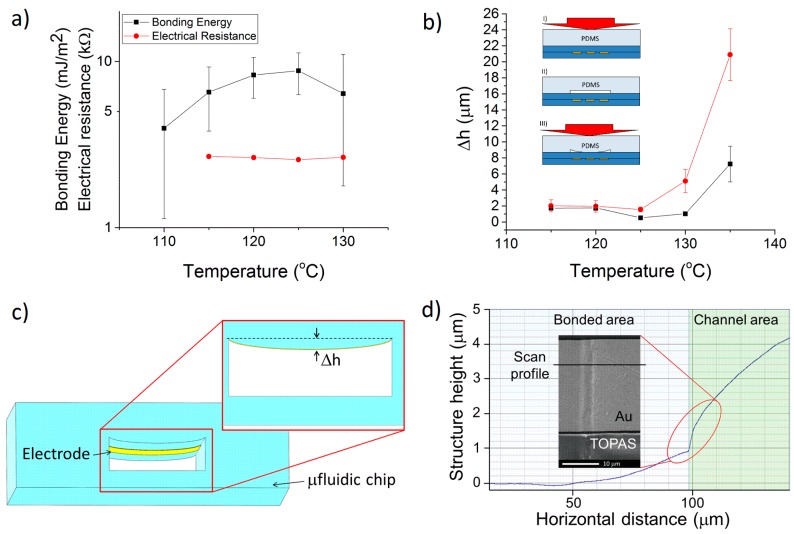
(**a**) Bonding Energy (*G*_f_) and electrical resistance (*R*_TB_) as a function of temperature for thermal bonding (TB) samples with *d*_g_ = 0. *G*_f_ and *R*_TB_ respectively have a maximum and minimum value at 125 °C; (**b**) Height Δ*h* vs. temperature for TB. An indent in the polydimethylsiloxane (PDMS) layer used to bond the electrodes was made to release the pressure on the area including the electrodes (details II–III) with respect to when the bonding was performed with no indent (detail I). The profilometric measurements on chips bonded with either the flat PDMS (red curve, detail I) or the indented PDMS (black curve, details II, III) show lower values of Δh in the electrode area when the bonding was done using the indented PDMS; (**c**) Scheme of substrate and electrode deformation in 3D after TB and 2D detail defining the height Δ*h*; (**d**) Profilometry/scanning electron microscopy (SEM) image of an Au electrode after TB and mechanical delamination of the chips. TB causes the creation of a height variation Δ*h* and an indentation of the electrodes. The 210 nm-thick electrode, bonded for 15 min at 135 °C with a force of 0.5 kN, was not embedded in the substrate (*d*_g_ = 0).

All resistance measurements were performed on batches of three different chips, and the deviation was considered to be the relative standard error (RSE) of mean. Bonding energy of TB samples increased with growing bonding temperature, reaching a peak at 125 °C, and degrading for higher *T* (Figure 3a). The first resistance measurements of TB systems with no grooves (*d*_g_ = 0) resulted in resistances between 50 and 100 kΩ. The effect of TB on the thin film electrode was also studied after delamination. Results showed that the applied load at the channel interface could cause a height difference Δ*h* (Figure 3b–d), and thus excess strain on the metal thin film. Since TOPAS 5013 has a heat deflection temperature (THD) of 130 °C [30], and since no significant deformation of nanochannels was previously found during bonding at 125 °C [33], tensile stress causing ruptures is the most likely cause of poor electrode conductance at low temperature bonding, while deformation caused by heat is the predominant effect when the temperature reaches the THD. 

Although measurements of mechanical strain were not performed, the rupture of free-standing Au thin films occurs at elongations of 3%–4% and with loads of 0.5 kN [34]. Such parameters are compatible with the optimal ones used for the TM of our thin-film electrodes.

In order to alleviate the pressure during TB, a PDMS layer with a 200 μm indent in the electrode area was used. This lowered the pressure during bonding, and thus to decreased the stress. With this modification, the resistance values were lowered to about 2.5 kΩ (Figure 3a). Measurements of Δ*h* on different chips with and without the indent show how the values of the height difference could be lowered with this method (Figure 3b). To further relieve the stress on the electrodes, the fabrication process was modified by embedding the electrodes inside grooves in the substrate before bonding [28].

**Figure 4:**
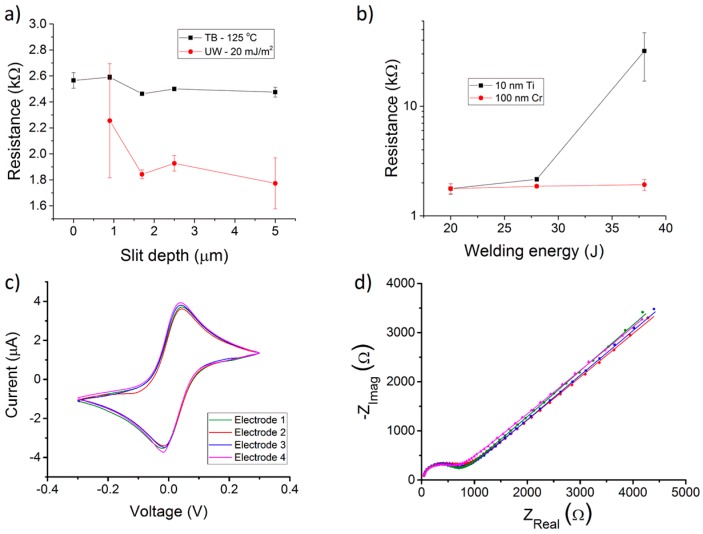
(**a**) Electrical resistance of thermal bonding (TB) and ultrasonic welding (UW) chips vs. groove depth. The electrodes have a 10 nm Ti adhesion layer. The TB is performed at *T* = 125 °C, 1 kN, for 10 min, while UW is with a welding energy of 20 mJ/m^2^. The values for TB are between 15% and 40% higher than the ones obtained with UW; (**b**) Electrical resistance of UW electrodes made with both 10 nm Ti and 100 nm Cr adhesion layers as a function of welding energy. In both cases, the adhesion layer was coated with a 200 nm-thick Au layer. Chips with a 10 nm Ti adhesion layer were disconnected when welded with 38 J, and leaked when welded with 15 J; (**c**) Cyclic voltammetric (CV) and (**d**) electrochemical impedance spectroscopic (EIS) characterization of Au electrodes in one of the chips bonded by UW. The superimposed voltammograms (**c**) and Nyquist plots (**d**) show the reproducible behavior of the four WEs. Details of the experimental conditions are given in the Materials and Methods section, and representative CV and EIS data of TB samples are provided in the supplementary information.

Although the resistance reached lower values in the case of deeper grooves (Figure 4b), we could not register a significant improvement. Since Δh is smaller than *d*_g_ in the majority of cases, this is a clear indication of the fact that the stress is caused primarily by the bending of the substrate with the embedded electrodes rather than from the direct pressing of the microfluidic channels on the electrodes. After process optimization, we chose 125 °C, 1 kN (500 kPa) for 10 min as our optimal bonding recipe. To benchmark our findings, we compared our results to the ones obtained by Illa et al. [29], where TB of COC was performed at a temperature of 120 °C for 15 min at a pressure of 5 MPa (8 kN over 16 cm^2^) on Au electrodes with a 200 nm thickness. The flatness of our substrates allows the bonding at 120 °C with lower pressures (around 2.5 MPa) and lower bonding times (5 min), thus causing a lower stress on the metal electrodes. 

The electrode performance and the high resistance measured in our case are probably due to the sharper edges of the fluidic chips with respect to the ones of the systems presented by Illa et al. The reason for the sharper edges is in the higher filling properties of IM with respect to the ones of the imprinting technique used by Illa et al. This is clearly visible from the section of the COC chip (Figure 2 of reference [29]) where the edges of the chip appear rounded, thus relieving the pressure on the Au electrodes at the channel interface. Achieving such edge rounding in IM chips would further improve TB, lower the electrical resistance and allow fabrication in larger numbers than the one obtainable with imprinting. The electrical resistance measurements of the UW chips were performed as a function of the groove depth (Figure 4a) and of delivered energy (Figure 4b). The resistance values are lower than the ones obtained with TB, but were still more than 15 times higher than the solution resistance. This indicates that the UW is also delivering stress to the samples and increasing the resistance. A partial solution to this issue was found by making a thicker electrode adhesion layer (100 nm Cr instead of 10 nm Ti) that stiffened the electrodes. Figure 4b shows a comparison of resistance between electrodes with the 10 nm Ti and the 100 nm Cr adhesion layer. For electrochemical experiments, electrodes deposited in 5 μm-deep grooves with a 100 nm Cr adhesion layer and bonded with energy of 20 J were used.

### 3.2. Electrochemical Results

The samples bonded with the optimized recipes were used for the electrochemical measurements: the UW-bonded chips showed good performance based on both CV and EIS characterization (Figure 4c,d). The CV performance was evaluated in terms of peak currents (*I*_p_) and peak separation potentials (Δ*E*_p_) as shown in Table 2. The highest values of *I*_p_ and lowest values of Δ*E*_p_ were obtained using substrates with electrodes that were embedded in 5 μm grooves. The optimized welding parameters (*F*_u_ = 350 N, *E* = 20 mJ/m^2^, *A* = 50%) used to bond the chips are considerably lower than the ones previously reported for electrodeless chips replicated from mechanically-milled stamps [21]. Moreover, we also demonstrate a uniform welding of energy directors over an area with an extension of about 36 mm (Figure 1). Although measurements of the curvature of the IM structures were not performed, the latter does not influence the welding, even with energy directors as low as 8 μm. The method described here is ideal for systems with an inter-electrode distance below approximately 400 μm. With higher electrode–electrode distances, it is possible to eliminate the height Δ*h* (Figure 3c) by making housings on the electrode substrates where the energy directors can be designed for gapless welding method [16,21].

Results in Table 2 show that the UW chips performed better than the TB chips in terms of both *I*_p_ and Δ*E*_p_, the latter being close to the ideal value of 59 mV [35] in the case of UW chips. The performance of the UW chips was also evaluated by acquiring impedance spectra that were fitted using Randles’ equivalent circuit model. The electrode performance was determined based on the reproducibility of the key parameters of the equivalent circuit model; i.e., the charge transfer resistance (*R*_ct_) and double layer capacitance (*C*_dl_) [32]. The determined chip-to-chip variation of *R*_ct_ and *C*_dl_—expressed as RSE of mean for three chips—was 0.7% and 5.2%, respectively. On-chip variation of *R*_ct_ and *C*_dl_ for the spectra shown in Figure 4d, expressed as relative standard deviation (RSD), was 1.0% and 5.9%, respectively. 

The comparison between UW and TB chips (presented above) indicates that UW yields chips with clearly better electrochemical performance than the TB ones, in terms of more precise redox potentials and lower series resistance. Furthermore, even when using the best TB bonding parameters, each fabrication batch had several chips with several non-functional electrodes. In contrast, for chips bonded using UW, electrode functionality yield was close to 100%, with more than 100 chips fabricated.

## 4. Conclusions

We have presented device integration using a process that involves the ultrasonic welding (UW) of IM polymer substrates with embedded electrodes to substrates containing a microfluidic network. Optimization of thermal bonding (TB) and UW was performed by measuring the electrical resistance of the electrodes. To lower the resistance, bonding temperatures and pressures were varied in TB. Changes in welding energy, as well as in electrode seed layer thickness, improved the electrode performance on UW samples. In both UW and TB, embedding of the electrodes inside grooves contributed to a lowering of the resistance. The electrochemical performance of electrodes embedded in polymer substrates that were subjected to either UW or TB was evaluated using cyclic voltammetry (CV) and electrochemical impedance spectroscopy (EIS). Based on resistance measurements and CV characterization, electrodes on UW-bonded chips had significantly better electrochemical performance in comparison to TB chips. CV evaluation also showed that in a batch of fabricated UW chips, close to 100% of electrodes were fully functional, whereas in a batch of TB chips bonded using the optimized parameters, several chips had totally non-functional electrodes. Moreover, EIS characterization demonstrated that electrodes on UW chips had low chip-to-chip variation of both charge transfer resistance and double layer capacitance. The presented fast integration process using UW provides fabrication throughput that matches injection molding and maintains the quality of thin-film electrodes. This opens up interesting possibilities to scale up production of microfluidic systems for electrochemical applications relying on both voltammetric techniques and EIS. Finally, the high fabrication throughput allowed the use of the chips for educational purposes: during a summer school, students with no previous training were able to perform electrochemical experiments that involved the repetition of previously published assays, such as the measurement of yeast redox activity and the detection of dopamine [36,37,38].

## Figures and Tables

**Table 1:** Parameter values for ultrasonic welding (UW) and thermal bonding (TB). The pressures (P) for TB are calculated considering that the force F_t_ insisted on a 2-inch wafer area.

UW		TB	
*F*_u_ (N)	350	*T* (°C)	110–135
*E*_UW_ (J)	20–38	*F*_t_ (kN)/P (MPa)	1–10/0.49–4.93
*A* (%)	50	*t* (min)	5–20
*d*_g_ (μm)	0.9/1.7/2.5/5	*d*_g_ (μm)	0/0.9/1.7/2.5/5

**Table 2:** Reduction and oxidation peak parameters based on cyclic voltammograms of the four working electrodes (average ± standard deviation) of a chips bonded with thermal bonding (TB) and ultrasonic welding (UW).

	TB		UW	
	Red	Ox	Red	Ox
*I*_p_ (μA)	3.17 ± 0.06	−3.27 ± 0.05	3.74 ± 0.07	−3.52 ± 0.06
Δ*E*_p_ (mV)	180 ± 7		67.5 ± 1	

## Supplementary Material

The following are available online at: http://www.mdpi.com/1424-8220/16/11/1795/s1, Figure S1: Representative data for (a) electrical cyclic voltammetry (CV) and (b) electrochemical impedance spectroscopic (EIS) characterization of Au electrodes in a chip thermally bonded at T = 125 °C, F = 1 kN, t = 10 min.
