# Supplementary material for: Comparison of Ultrasonic Welding and Thermal Bonding for the Integration of Thin Film Metal Electrodes in Injection Molded Polymeric Lab-on-Chip Systems for Electrochemistry
Source: Sensors (Basel). 2016 Oct 27;16(11):1795. doi: 10.3390/s16111795 (PMC5134454; doi:10.3390/s16111795)
Supplement: Supplementary file 1 [file sensors-16-01795-s001.pdf]

Supplementary Materials: Comparison of Ultrasonic Welding and Thermal Bonding for the Integration of Thin Film Metal Electrodes in Injection Molded Polymeric Lab-on-Chip Systems for Electrochemistry

Marco Matteucci, Arto Heiskanen, Kinga Zór, Jenny Emnéus and Rafael Taboryski

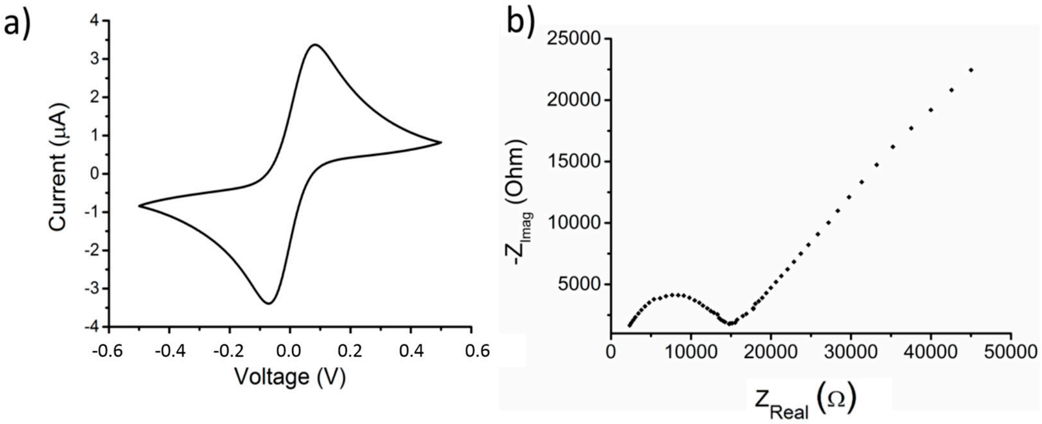

Figure S1. Representative data for (**a**) electrical cyclic voltammetry (CV) and (**b**) electrochemical impedance spectroscopic (EIS) characterization of Au electrodes in a chip thermally bonded at T = 125 °C, F = 1 kN, t = 10 min.
